# Supplementary material for: Voriconazole Eye Drops: Enhanced Solubility and Stability through Ternary Voriconazole/Sulfobutyl Ether β-Cyclodextrin/Polyvinyl Alcohol Complexes
Source: Int J Mol Sci. 2023 Jan 25;24(3):2343. doi: 10.3390/ijms24032343 (PMC9917179; doi:10.3390/ijms24032343)
Supplement: Supplementary file 1 [file ijms-24-02343-s001.zip › ijms-2188185-supplementary.pdf]

International Journal of *Molecular Sciences*

MDPI

Supplementary Materials

Voriconazole Eye Drops: Enhanced Solubility and Stability through Ternary Voriconazole/Sulfobutyl Ether β-cyclodextrin/Polyvinyl Alcohol Complexes

Hay Man Saung Hnin Soe [1], Khanittha Kerdpol [2], Thanyada Rungrotmongkol [2,3], Patamaporn Pruksakorn [4], Rinrapas Autthateinchai [4], Sirawit Wet-osot [4], Thorsteinn Loftsson [5] and Phatsawee Jansook [1,*]

1 Faculty of Pharmaceutical Sciences, Chulalongkorn University, 254 Phyathai Road, Pathumwan, 10330 Bangkok, Thailand; haymansaunghninsoe@gmail.com
2 Center of Excellence in Biocatalyst and Sustainable Biotechnology, Department of Biochemistry, Faculty of Science, Chulalongkorn University, 254 Phyathai Road, Pathumwan, 10330 Bangkok, Thailand; khanittha.view@gmail.com (K.K.), thanyada.r@chula.ac.th (T.R.)
3 Program in Bioinformatics and Computational Biology, Graduate School, Chulalongkorn University, 10330 Bangkok, Thailand; thanyada.r@chula.ac.th
4 Department of Medical Sciences, Ministry of Public Health, Tiwanon Road, 11000 Nonthaburi, Thailand; patamaporn.p@dmsc.mail.go.th (P.P.), rinrapas.a@dmsc.mail.go.th (R.A.), sirawit.w@dmsc.mail.go.th (S.W.)
5 Faculty of Pharmaceutical Sciences, University of Iceland, Hofsvallagata 53 IS-107 Reykjavik, Iceland; thorstlo@hi.is
* Correspondence: phatsawee.j@chula.ac.th; Tel.: +662-218-8273

Citation: Soe, H.M.S.H.; Kerdpol, K.; Rungrotmongkol, T.; Pruksakorn, P.; Autthateinchai, R.; Wet-osot, S.; Loftsson, T.; Jansook, P. Voriconazole eye drops: enhanced solubility and stability through ternary voriconazole/sulfobutyl ether β-cyclodextrin/polyvinyl alcohol complexes. *Int. J. Mol. Sci.* **2023**, *24*, x. https://doi.org/10.3390/xxxxx

Academic Editor(s): Miguel A. Esteso; Carmen M. Romero

Received: 12 January 2023
Revision: 21 January 2023

Accepted: 23 January 2023
Published: date

Publisher's Note: MDPI stays neutral with regard to jurisdictional claims in published maps and institutional affiliations.

Copyright: © 2023 by the authors. Submitted for possible open access publication under the terms and conditions of the Creative Commons Attribution (CC BY) license (http://creativecommons.org/licenses/by/4.0/).

International Journal of
Molecular Sciences

MDPI

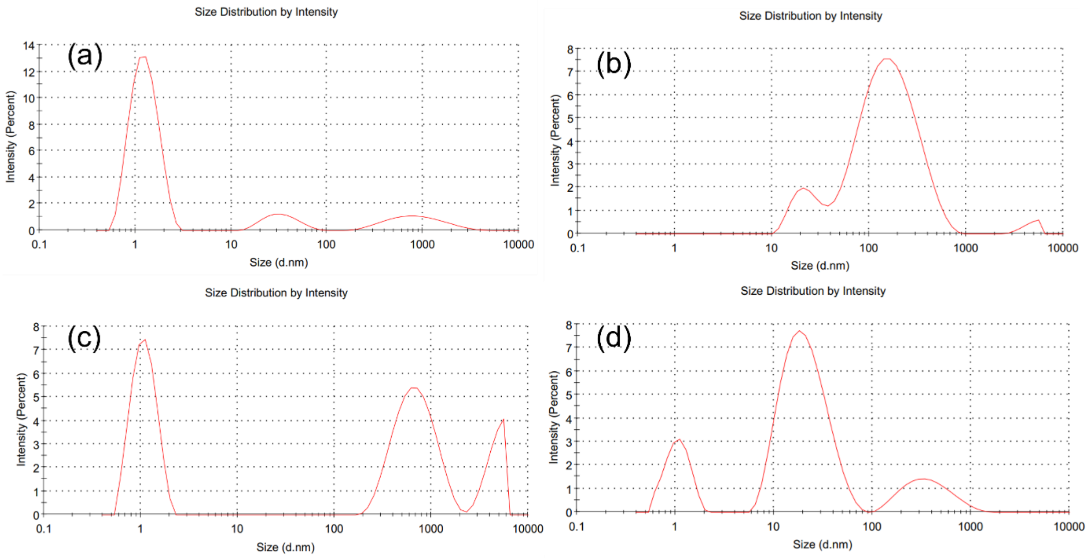

Figure S1. Aggregate size and size distribution by intensity of binary VCZ/SBEβCD and ternary VCZ/SBEβCD/polymer complex aggregates determined by DLS technique at 25 °C; (a) VCZ/SBEβCD, (b) VCZ/SBEβCD/CS, (c) VCZ/SBEβCD/HA and (d) VCZ/SBEβCD/PVA

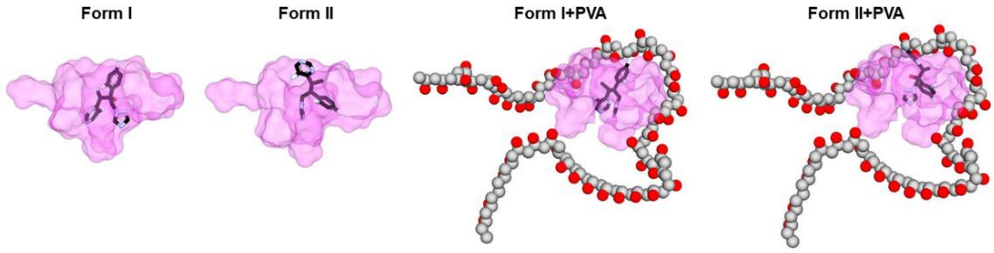

Figure S2. Two different forms of the VCZ/SBEβCD and VCZ/SBEβCD/PVA inclusion complexes generated from molecular docking protocol used as the initial models for MD simulations..

International Journal of *Molecular Sciences*

MDPI

Table S1. Release kinetic model fitting of VCZ from PVA-L solution and PVA-H hydrogel.

Formulation	Zero order		First order			Higuchi		Hixson-Crowell		Korsmeyer-Peppas		
	K_0[a]	R^2	K_1[b]	n	R^2	K_H[c]	R^2	K_{Hc}[d]	R^2	n[f]	K[e]	R^2
PVA-L solution	1.0439	0.8028	-0.0067	1.9506	0.8710	8.7126	0.9318	0.0208	0.1762	0.7446	0.6391	0.9120
PVA-H hydrogel	0.7511	0.8267	-0.0041	1.9702	0.8628	6.2270	0.9469	0.0136	0.8512	0.7996	0.4040	0.9402

[a]K_0: zero order release constant; [b]K_1: first order release constant; [c]K_H: Higuchi constant; [d]K_{Hc}: Hixson-Crowell constant; [e]K: Korsmeyer-Peppas constant; [f]n: the diffusion or release exponent
